# Supplemental Nicotinic Acid Elevates NAD^+^ Precursors in the Follicular Fluid of Mares

**DOI:** 10.3390/ani12111383

**Published:** 2022-05-27

**Authors:** Charley-Lea Pollard, Zamira Gibb, Jennifer Clulow, Agustin Ruiz, Alecia Sheridan, Mohammad Bahrami, Aleona Swegen, Christopher G. Grupen

**Affiliations:** 1 Sydney School of Veterinary Science, Faculty of Science, The University of Sydney, Camden, NSW 2570, Australia; christopher.grupen@sydney.edu.au; 2 Priority Research Centre for Reproductive Science, University of Newcastle, Callaghan, NSW 2308, Australia; zamira.gibb@newcastle.edu.au (Z.G.); alecia.sheridan@newcastle.edu.au (A.S.); mo.bahrami@newcastle.edu.au (M.B.); aleona.swegen@newcastle.edu.au (A.S.); 3 Scone Equine Hospital, Scone, NSW 2337, Australia; jeclulow@csu.edu.au; 4 Newcastle Equine Rehabilitation and Reproduction Centre, Luskintyre, NSW 2321, Australia; agustin.ruiz@newcastleequine.com.au; 5 Nuffield Department of Women’s and Reproductive Health, University of Oxford, Oxford OX1 2JD, UK

**Keywords:** equine, follicular fluid, mare, NAD, nicotinic acid, mass spectrometry

## Abstract

**Simple Summary:**

Miscarriage and embryonic death have been associated with a deficiency in NAD^+^ resulting from a lack of dietary niacin in women and mice. Mares often suffer from high rates of early embryonic loss, but the dietary requirement of niacin in pregnant mares remains unknown. The aim of this study was to determine the effect of supplementing nicotinic acid, a form of niacin often supplemented in horse diets, on the elevation of other NAD^+^ precursors in the blood and follicular fluid of mares. We orally administered nicotinic acid to mares over consecutive days and collected blood and follicular fluid at the end of the feeding period. The results show that multiple examined NAD^+^ precursors were elevated in the follicular fluid of mares at the end of the feeding period, which we propose will aid in reducing early embryonic loss in the mare by promoting good quality oocytes.

**Abstract:**

A deficiency in NAD^+^ has previously been linked with increased occurrences of congenital abnormalities and embryonic death in humans and mice. Early embryonic death is a major factor involved in pregnancy loss in mares, and very little is known regarding the NAD^+^ requirements for optimum reproductive function in horses. The aim of this study was to determine the effect of supplementing the diet of mares with nicotinic acid (NA) on the composition of NAD^+^ metabolites in the blood and follicular fluid. Vehicle alone or NA (3 g per os) were administered to seven mares over a minimum of 3 consecutive days during the follicular phase of the oestrous cycle. Blood samples were collected immediately prior to supplemental feeding and follicular fluid aspiration. Follicular fluid was collected from the dominant follicle through transvaginal ultrasound-guided aspiration. Blood and follicular fluid samples were processed and analysed by mass spectrometry. The concentration of nicotinamide mononucleotide (NMN) in the follicular fluid of NA-fed mares was 4-fold greater than that in the corresponding plasma and 10-fold greater than that in the follicular fluid of vehicle-fed mares. The concentrations of NA, nicotinamide (NAM) and nicotinuric acid (NUR) tended to be greater in the follicular fluid of NA-supplemented mares than in the corresponding plasma. The results show that NA supplementation increased the bioavailability of NAD^+^ precursors in the follicular fluid of the dominant follicle, which is proposed to better promote the maturation of good quality oocytes, especially in older mares.

## 1. Introduction

The reproductive efficiency of mares is much lower compared to other livestock species, largely due to the sacrifice in reproductive potential with the selection of stallions and broodmares based on their pedigree and athletic performance. Analysis of Thoroughbred and Standardbred breeding records reveals that pregnancy rates are greatly influenced by maternal age and reproductive status [1,2], and embryonic losses as high as 20–30% have previously been reported [3,4]. Repeated services or inseminations are often required, elevating the risk of stress and injury associated with breeding, which drastically increases the economic costs involved in achieving a successful pregnancy. The causes of embryonic losses are multifactorial and are largely attributed to defective embryos, inadequate maternal factors and external environmental factors [5,6]. Maternal nutrition is increasingly recognised as playing a role in embryonic loss [6,7] and may provide a non-invasive method of manipulating fertility.

**Figure 1:**
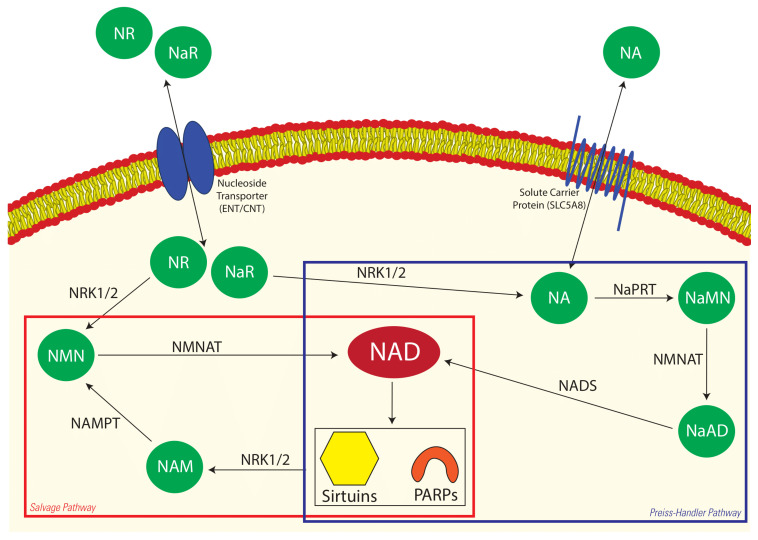
Two NAD^+^ biosynthetic pathways in the cell. NA enters the cell via facilitated diffusion through solute carrier proteins where it is converted to NaMN via NaPRT. NMNAT then converts NaMN to NaAD before it is converted to NAD^+^ via NADS. Sirtuin proteins and PARPs consume NAD^+^, which releases NAM. NAMPT then converts NAM to NMN, which is then recycled back into NAD^+^ by NMNAT. NaR and NR enter the cell through nucleoside transporter proteins where they feed into the Preiss–Handler and salvage pathways, respectively. The two NAD^+^ pathways are highlighted in their respective boxes. The metabolites examined in this study are represented in green, and NAD^+^ is the end product of each pathway, represented in red. NA—nicotinic acid, NaMN—nicotinic acid mononucleotide, NaAD—nicotinic acid adenine dinucleotide, NAD—nicotinamide adenine dinucleotide, NAM—nicotinamide, NMN—nicotinamide mononucleotide, NR—nicotinamide riboside, NaR—nicotinic acid riboside, PARPs—poly-ADP-ribose polymerases, NRK1/2—nicotinamide riboside kinase1/2, NaPRT—nicotinic acid phosphoribosyltransferase, NMNAT—nicotinamide mononucleotide adenylyltransferase, NADS—NAD synthase, NAMPT—nicotinamide phosphoribosyltransferase.

Nicotinamide adenine dinucleotide (NAD^+^) is an essential cofactor involved in many cellular processes, including energy metabolism, cell survival, DNA repair and, more recently, aging [8,9]. Sirtuins (SIRTs) and poly-ADP-ribose polymerases (PARPs) are NAD^+^-dependent proteins and enzymes that consume NAD^+^ in their various reactions, depleting the cellular NAD^+^ pools. As such, there is a need to constantly replenish NAD^+^ stores to keep these processes functioning within the cell. NAD^+^ is synthesised through three methods: the metabolism of dietary niacin (nicotinic acid, vitamin B3) [10] in the Preiss–Handler pathway; through the conversion of the amino acid tryptophan [11] via de novo synthesis; and through the salvage of nicotinamide (NAM; Figure 1). NAD^+^ is essential for normal embryo development; genetic mutations disrupting NAD^+^ synthesis lead to congenital malformations in humans [12], and embryonic death and birth defects are associated with NAD^+^ deficiency in mice [13,14]. Interestingly, when NAD^+^-deficient and maternally aged mice were supplemented with nicotinamide mononucleotide (NMN) in drinking water, the oocyte quality and embryo development rates were restored [13,14].

While a dietary niacin intake of 12 mg/kg dry matter is recommended for adult horses [15], there have been few studies investigating the metabolism of niacin in horses and none examining the requirement for niacin in mares during pregnancy. The biosynthesis of water-soluble vitamins, including niacin, by the hindgut microbiota is presumed to meet the requirement for niacin when dietary intake is insufficient [16], and as such, there are no current recommendations for dietary niacin requirements in horses [17]. Previous studies have revealed no effect of the oral supplementation of nicotinic acid on niacin status in working Thoroughbred geldings as assessed by an examination of the NAD:NADP ratio in red blood cells [18], and that removal of niacin from the diet had no effect on growth rates [19]. However, NAD^+^ is an intracellular metabolite with a short half-life and is rapidly degraded on cell lysis [20,21], which may have contributed to the lack of differences in the NAD:NADP ratio in red blood cells, particularly considering that NAD^+^ is unstable in whole blood [22]. The small intestine is the primary absorption site for niacin [23], and as this site is upstream of the caecum where the production of niacin by microbes occurs [24], it is possible that microbial niacin was not available for absorption in these studies.

Recently, a mass spectrometric analysis of mare serum and urine samples following nicotinic acid supplementation at a supraphysiological dose (5 g) showed that the bioavailability of NAD+ precursors was significantly increased, with a sustained elevation of nicotinic acid adenine dinucleotide (NaAD) levels, indicating that the NAD^+^ levels were boosted within the cellular compartments [25]. Furthermore, studies investigating dietary niacin requirements in humans have revealed that women require more niacin than men [26,27,28,29], particularly during pregnancy [30]. Given the importance of dietary niacin for fertility in women and the influence of NAD^+^ precursors in female mice, we wished to investigate the potential for improving reproductive outcomes via nicotinic acid supplementation in mares. As studies investigating niacin metabolism in horses have not focussed on mare reproductive status [18,19], the aim of this study was to determine whether orally administered nicotinic acid could lead to increased concentrations of NAD^+^ metabolites in the follicular fluid (FF) of mares, opening a potential avenue for improving fertility in ageing mares of high genetic merit.

## 2. Materials and Methods

### 2.1. Animals

Six Standardbred mares between 13 and 17 years of age and one 7-year-old Thoroughbred mare were included in the study. All mares had a body condition score of 5 or 6 on the nine-point Henneke Body Condition Scoring System [31] and were in good physical health with a body weight range of 432.94 to 606.21 kg (mean ± S.D.; 494.75 ± 55.75 kg). The mares were housed in a large paddock and maintained on a forage diet (comprising native and improved pasture and some lucerne hay). A clinical examination was performed on all mares prior to their inclusion in the study.

### 2.2. Study Design

A crossover design was employed in this study, and all mares served as their own control. The clearance rate of nicotinic acid (NA) in the ovary and follicular fluid remains unknown, so all mares were first treated with the vehicle control followed by NA after a 12-day washout period in order to prevent possible residual effects of the NA treatment.

### 2.3. Supplemental Feeding and Blood Collections

**Figure 2:**
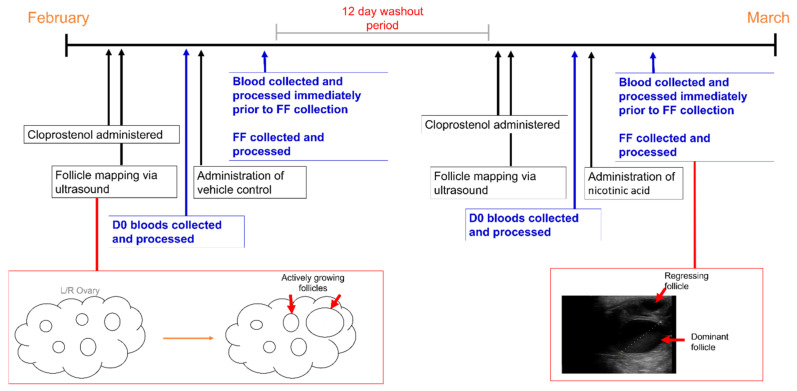
A timeline of events for the study period. The study was conducted in February and March, towards the end of the equine breeding season in Australia. Cloprostenol was administered to all mares to synchronise oestrus. Follicles were mapped daily via transvaginal guided ultrasound to identify regression of the corpus luteum and the beginning of active follicle growth. Blood samples were collected prior to any treatments being administered, followed by the administration of the vehicle control or nicotinic acid. Once a dominant follicle (≥25 mm in diameter) was identified, feeding ceased, and blood and follicular fluid were collected and processed approximately 16 h after the final feeding.

A timeline covering the study period and all major sample collection points and procedures is shown in Figure 2. Mares were routinely scanned using transrectal ultrasound and were administered 1 mL Cloprostenol (250 µg/mL, Estrumate) to facilitate oestrus synchronisation. Prior to the commencement of the first vehicle and NA treatments, whole blood was collected from the jugular vein via 20-gauge needles into Vacutainer™ blood sampling tubes containing 3.2% sodium citrate (Livingstone; BCTCIT). Upon collection, blood samples were immediately spun at 1500× *g* for 2 min. Aliquots (100 µL) of plasma were snap-frozen in triplicate and stored at −80 °C until analysis. For the control (vehicle treatment), all mares were orally administered 10 mL of fresh apple sauce using a 50 mL dosing syringe. The day of dominant follicle identification (at least 25 mm in diameter) determined the number of consecutive days the mares received the vehicle treatment to ensure the supplement was directed to an actively growing follicle with a viable oocyte at concentrations that were less likely to invoke detrimental effects. As such, each mare was fed for a minimum of 4 consecutive days. Blood samples were collected immediately prior to sedation and FF aspiration (approximately 16–18 h after the last vehicle treatment) and processed as above. For the NA treatment, following a 12-day washout period, all mares were orally administered 3 g of NA mixed thoroughly in 10 mL of fresh apple sauce for a minimum of 3 consecutive days. This dose was chosen based on the findings of our previous study in which approximately 3.5 g of NA was absorbed, metabolised and distributed to cells via the circulation [25] and is about 30 times greater than the recommended daily requirement [15]. As before, the day of dominant follicle identification determined the number of consecutive days the mares received the NA treatment. Blood samples were collected immediately prior to sedation and FF aspiration (approximately 16–18 h after the last NA treatment) and processed as above.

### 2.4. Follicular Fluid Aspiration and Mare Husbandry

The day before FF aspiration, mares were examined via transrectal ultrasound to ensure the presence of a dominant follicle (>30 mm). If a dominant follicle was present, mares were administered 1 mL of deslorelin acetate (1.25 mg/mL IM) 18–24 h prior to FF aspiration to stimulate follicular growth. On the day of FF aspiration, a clinical examination was performed prior to the administration of medication to ensure that the mares were fit for the procedure. Mares were pre-medicated with procaine penicillin G (22 mg/kg IM) and gentamicin sulphate (6.6 mg/kg IV) in addition to flunixin meglumine (flunixin injection, 1.1 mg/kg IV) prior to the transrectal ultrasound. The mares were sedated using detomidine hydrochloride (Dormasedan, 10 mg/mL; 0.005–0.015 mg/kg) and butorphanol tartrate (Butordyne, 10 mg/mL; 0.01–0.02 mg/kg). In addition, hyoscine butyl-bromide (Buscopan, 15–20 mL) was administered to facilitate smooth muscle relaxation and ease of ovarian manipulation. The perineum was prepared aseptically, and the transvaginal ultrasound probe was inserted to allow the visualisation of the ovary through the vaginal fornix. Follicular fluid was collected from the dominant follicle via transvaginal guided ultrasound with a 12-gauge double-lumen needle (Minitube; 19009/3105) connected to a vacuum pump (Cook Medical, Australia; K-MAR-5200). Samples were collected approximately 16–18 h after the last NA treatment. Follicular fluid was aspirated into sterile 50 mL Falcon tubes (Bacto, Australia; 352098). Aliquots (100 µL) of FF were snap-frozen in triplicate and stored at −80 °C until analysis.

### 2.5. Chemicals and Reagents

All chemicals and reagents were purchased from Sigma Aldrich (Castle Hill, Australia) unless otherwise stated. Nicotinic acid (NA; N4126; purity ≥ 98%), nicotinamide (NAM; 72340; purity ≥ 99.5% HPLC), nicotinuric acid (NUR; N4751; purity ≥ 98%) and 3-quinolinecarboxylic acid (3QCA; 177148; purity 98%) were sourced as standards. Nicotinamide mononucleotide (NMN), nicotinamide adenine dinucleotide (NAD), nicotinic acid adenine dinucleotide (NaAD) and nicotinic acid mononucleotide (NaMN) were readily available in 1 mM stock solution. Sample extraction and preparation chemicals included acetonitrile (ACN; A955), methanol (MeOH; A456) and Optima LC/MS water (W6; Fisher Chemical; North Ryde, Australia), formic acid (85178; LC-MS grade; purity ≥ 99%; Thermo Scientific), hexane (Science Supply Australia; Unilab; 251-2.5 L; Mitcham, Australia) and ammonium hydroxide solution (NH_4_OH; 44273; 28–30% in H_2_O).

### 2.6. Solid-Phase Extraction

Isolute solid-phase extraction columns (SCX 500 mg/3 mL; Shimadzu Scientific Instruments (Oceania); BIOT-530-0050-B; Rydalmere, Australia) were prepared as previously described [22]. In brief, columns were preconditioned with 2 mL of MeOH followed by 1 mL of 1% formic acid. Plasma and FF samples were applied to the column and washed with 2 mL of ACN, then with 2 mL of MeOH and finally with 2 mL of hexane. The samples were then eluted with 3 mL of MeOH containing 2% NH_4_OH and the samples were dried by vacuum centrifugation. The plasma and FF samples were then reconstituted in 50 µL of LCMS water before being applied to the HPLC column.

### 2.7. Mass Spectrometry Instrumentation and Conditions

Liquid chromatography and mass spectrometry were carried out on an Agilent 1260 Infinity pump coupled to a QTRAP5500 (AbSCIEX). The chromatographic separation of NAD^+^ metabolites was achieved using an XBridge Beh Amide column (130Å, 3.5 µm, 201 mm × 100 mm; Waters, Australia) at a flow rate of 0.2 mL/min with a total run time of 30 min. The mobile phase consisted of ACN with 20 mM ammonium acetate and 20 mM NH_4_OH (A) and 100% ACN (B). The initial composition of the mobile phase was 15% A and 85% B, which was ramped up to 30% A after 10 min and held for 3 min before being increased again to 70% A for 5 min and 30 s before returning to initial levels and being maintained for the duration of the run. An injection volume of 2.5 µL was used for all samples. The ion source voltage was set at 4500 V, the capillary temperature was set at 350 °C and the pressure was maintained at 45 bar. Data were acquired and analysed with Analyst 1.6.2 and MultiQuant software, respectively.

### 2.8. Statistical Analysis

Data were subjected to a log transformation to standardise the residuals and analysed using R (version 3.5.3; 2019; R Core Team; Vienna, Austria). The data were subjected to a residual maximum likelihood regression using the lmerTest package (version 3.1-2) with treatment as a factor and horse as a random error. A linear regression via the lmerTest package was used to determine if the differences between treatments were due to differences between individual mares and the two separate sampling periods in the absence of a double crossover design. A multiple comparisons test using the emmeans statistical package (version 1.4.7) was used to determine the samples that differed significantly. Data are presented as the untransformed means ± S.E.M., and a *p*-value of < 0.05 was considered significant.

## 3. Results

### 3.1. Metabolite Concentrations in Plasma

**Figure 3:**
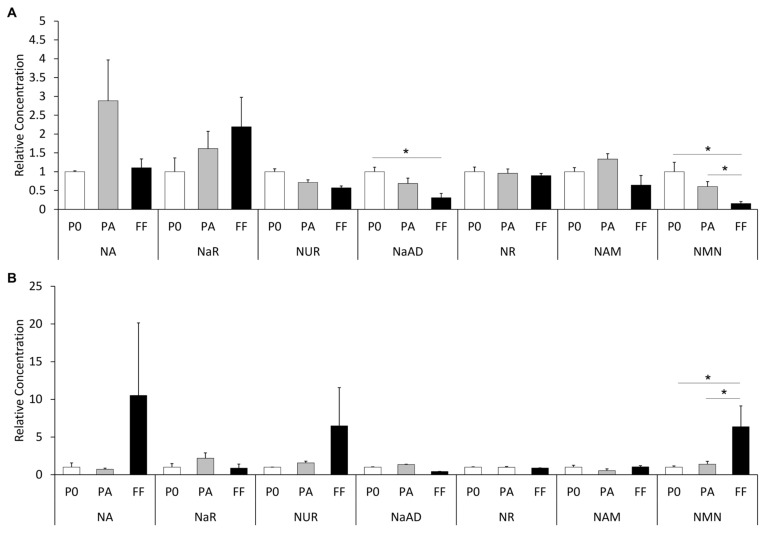
The relative concentrations of NAD^+^ metabolites in the plasma and follicular fluid of (**A**) vehicle-fed and (**B**) NA-fed mares. Data are presented as a proportion of the baseline plasma prior to administration of the first feed (P0). PA–plasma at time of follicle aspiration, FF–follicular fluid. * indicates a statistical difference of *p* < 0.05.

In the plasma of mares fed the vehicle treatment, the concentrations of all metabolites did not differ between the plasma at the time of follicle aspiration and that prior to the first vehicle feed being administered (*p* > 0.05; Table 1 and Figure 3A). Similarly, in the plasma of mares fed the NA treatment, the concentrations of all the NAD^+^ precursors at the time of follicle aspiration were similar to those prior to the first NA feed being administered (*p* > 0.05; Table 1 and Figure 3B).

Plasma metabolite concentration differences were apparent at both sampling time points between the vehicle- and NA-fed groups. Prior to the first administrations, the concentrations of NaAD, NR and NUR were significantly greater in the vehicle-fed mares than in the NA-fed mares (*p* < 0.05; Table 1 and Figure 3). Likewise, at follicle aspiration, the plasma levels of NaAD and NR were significantly greater in the vehicle-fed mares (*p* < 0.05; Table 1 and Figure 3A). In contrast, the plasma concentration of NAM prior to the first administrations was increased in the NA-fed mares compared with that in the vehicle-fed mares (*p* < 0.001; Table 1 and Figure 3), while the concentration at follicle aspiration was not different between the vehicle- and NA-fed mares (*p* > 0.05; Table 1 and Figure 3).

### 3.2. Metabolite Concentrations in the Follicular Fluid of Dominant Follicles

Comparing the FF samples of the vehicle-fed and NA-fed mares, the concentrations of several metabolites differed significantly. The FF levels of NMN and NAM in the NA-fed mares were 10- and 17-fold greater, respectively, compared with the vehicle-fed mares (*p* < 0.001 and 0.0001, respectively; Table 1 and Figure 3). Concomitantly, the FF level of NR in the vehicle-fed mares was 5-fold greater compared with the NA-fed mares (*p* < 0.0001; Table 1 and Figure 3). There were no differences in the follicular fluid concentrations of all other metabolites between the two treatments (*p* > 0.05; Table 1 and Figure 3).

In mares fed the vehicle treatment, the concentration of NMN in FF was lower (75% decrease) than that in the plasma collected at the time of follicle aspiration (*p* < 0.05; Table 1 and Figure 3A). The concentrations of NA, NaAD, NAM, NUR, NaR and NR in FF were similar to those in the plasma at follicle aspiration in the vehicle-fed mares (*p* > 0.05). In mares fed the NA treatment, the FF concentration of NMN was almost 5-fold higher than in the plasma at the time of follicle aspiration (*p* < 0.05). Conversely, the FF concentrations of the remaining NAD^+^ precursors (NA, NAM, NUR, NaR, NR and NaAD) did not significantly differ from those in the plasma at follicle aspiration.

## 4. Discussion

This study characterised the absorption and metabolism of nicotinic acid in the blood plasma and FF of mares following daily oral administration of NA during the follicular phase of the oestrous cycle. A mass spectrometric quantification of NAD^+^ precursors indicates that the supplemented NA was metabolised through the various NAD^+^ biosynthetic pathways such that the concentrations of some NAD^+^ precursors measured in the FF were similar to, or greater than, the concentrations found in plasma, thereby increasing the bioavailability of the associated metabolites to the follicular cells and maturing oocyte for NAD^+^ synthesis.

Of most significance was the elevation of NMN in the FF of NA-supplemented mares compared with the corresponding plasma sample at the time of FF aspiration. Conversely, in the vehicle-fed mares, the level of NMN in FF was much lower than that in the plasma. The elevation of NMN in the FF of NA-supplemented mares suggests that the administered NA was absorbed and metabolised by the cells of the dominant follicle via the salvage pathway, enhancing NAD^+^ synthesis during the final stages of follicle development. A previous study by Bertoldo et al. [13] showed that supplementing the drinking water of aged mice with NMN for 4 weeks boosted NAD^+^ biosynthesis, which resulted in improved oocyte quality and embryo development through the restoration of meiotic spindle assembly and a reduction in reactive oxygen species (ROS). Improvements in mitochondrial function, fertilising ability and developmental potential and reductions in DNA damage and ROS-induced apoptosis were also observed when aged mice were administered NMN via intraperitoneal injection for 10 consecutive days prior to ovulation [14]. Oocytes from women and mares of advanced age and equine oocytes harvested for in vitro maturation are known to display an increased incidence of spindle abnormalities that result in misaligned chromosomes [32,33,34], which likely results in aneuploidy and contributes to the age-related decline in mare fertility. Our findings demonstrate that the oral supplementation of NA during the follicular phase of the oestrous cycle in mares elevated the level of NMN present in the FF of the dominant follicle, providing tantalising evidence that this simple dietary intervention may boost NAD^+^ biosynthesis within the follicular compartment and enhance the viability of oocytes, especially in old mares.

The concentration of NA did not differ among the initial plasma samples and the plasma samples collected at the time of FF aspiration in both the vehicle-fed and NA-supplemented mares. In our previous study, we found that the concentration of NA in plasma peaked between 15 min and 1 h following the supplementation of NA [25]. Therefore, it is not surprising that the concentration of NA did not differ between the initial plasma and the plasma collected at the time of FF aspiration approximately 12–16 h after the final feed. Remarkably, the concentration of NA in the FF of NA-supplemented mares appeared to be 47-fold greater than that in the FF of vehicle-fed mares, but due to the high variation in the concentration of NA in the FF between mares, this difference was not significant. Recent studies have shown that NA supplementation of in vitro maturation media improves the quality of bovine and porcine oocytes [35,36,37]. The majority of niacin in the diet is absorbed in its bound form in pasture and is therefore unable to be absorbed through the intestinal wall [38]. Evidence suggests that microbial production of niacin is sufficient to meet the dietary needs of horses [16,18,19,24], but whether this is sufficient to meet the requirements for optimal reproductive performance is unknown.

Similar to the NA results, the concentration of NMN did not differ among the initial plasma samples and the plasma samples collected at the time of FF aspiration in both the vehicle-fed and NA-supplemented mares. Given that FF is partly an exudate of plasma and that the concentrations of both NA and NMN appeared to be markedly elevated in FF compared with the corresponding plasma sample, this suggests that these metabolites are selectively directed from the circulation to the growing dominant follicle. Alternatively, the metabolic activity of the cells within the dominant follicle resulted in the accumulation of NA and NMN in the FF. The salvage pathway is a constant loop whereby NAD^+^ is consumed by sirtuin proteins (SIRTs) and poly-ADP-ribose polymerases (PARPs), releasing NAM and O-acetyl-ADP-ribose (Figure 1) [11,39]. SIRTs are well known for deacetylating proteins that regulate numerous functions within the follicles, oocytes and embryos of several species, including horses [33,40]. Our findings indicate that supplemented NA was absorbed and metabolised through the Preiss–Handler pathway for the production of NAD^+^, and, following consumption by SIRTs and PARPs, NMN levels were elevated in the FF via the salvage pathway. That the level of NAM in FF was not elevated supports our suggestion that the salvage pathway was active in the developing follicle, resulting in the conversion of NAM to NMN. Upon ovulation, the contents of the dominant follicle are released into the oviduct, adding to the complex milieu that supports fertilisation and early embryonic cleavage. Improvements in mouse embryo development as a result of supplementing the embryo culture media with NMN in vitro have previously been demonstrated [13]. Hence, elevating the level of NMN in the oviductal environment has the potential to improve embryo development in mares.

There were no differences in the concentrations of NaR, NR or NAM in the FF or plasma samples at the time of FF aspiration in either treatment group. In this study, we did not detect NaMN or NAD^+^ in any of the samples. The latter is unsurprising; NAD^+^ is an intracellular cofactor [20]. However, the absence of NaMN is surprising, considering that we previously detected it in the plasma of mares at low concentrations [25] that returned to baseline levels at 22 h following the supplementation of NA. In the present study, the NA dose was lower than that used in the previous study, and samples were collected 12–16 h following the final feed as opposed to the time points of 0, 0.25, 0.5, 1, 2, 4, 6 and 22 h [25] so it is likely that the concentration was below the limits of detection in this study. Additionally, the finding that NaAD was not elevated in the plasma or FF following the dosing period was unexpected, given our previous findings. NaAD is considered the most sensitive biomarker for NAD^+^ metabolism [41] and was found to remain elevated in the plasma of mares for at least 22 h following the oral administration of a single supraphysiological dose of 5 g of NA [25]. In the present study, a NA dose of 3 g was used because the 5 g dose resulted in approximately 1.5 g of NA and associated metabolites being excreted in the urine, indicating it was excessive. Perhaps the lower NA dose facilitated more complete utilization of NaAD for the synthesis of NAD^+^.

At the commencement of each feeding treatment, when the first plasma sample was collected, the concentrations of NaAD, NAM, NMN, NR and NUR differed considerably between the two periods. This result was unexpected, as the mares were not supplemented with pellets or feed other than hay in the lead up to the first feeding period and during the 12-day washout period. The differences in environmental conditions leading up to each of the feeding periods may have contributed to the variations in metabolite concentrations seen in the plasma samples between the vehicle-fed and NA-fed groups. The trial was conducted over a 6-week period during February and March, towards the end of the breeding season in Australia. The occurrence of rain events differed between the two lead-up periods, leading to an abundance of grasses in pastures throughout the first feeding period, which could have increased the levels of NA and NR consumed in the forage. The niacin content of green grasses is reported to be 37 mg (ranging from 13 to 57 mg) per kg of digestible matter [42] and may change considerably under environmental stress [43]. While detailed analyses of the niacin content in grasses of native Australian pastures are lacking, differing niacin contents due to the changing environmental conditions may have contributed to the variation observed between the two sampling periods.

This study used a small number of animals, and there were large variations observed between animals in the concentrations of some metabolites, in particular NA, NUR and NaR. This suggests that the absorption and metabolism of NA varied considerably between individual mares, possibly due to differences in the mares’ metabolic demands and/or enzyme activities. These differences were not attributed to age in this study. Studies segregating a larger number of mares by age would provide more definitive results regarding the metabolic demands of aged mares. The collection of additional plasma samples along with urine sampling prior to and at the time of FF aspiration would have provided additional information on the metabolism and excretion of NA metabolites during the follicular phase of the oestrous cycle but was not possible due to procedural constraints. While a crossover study design where half of the mares were initially administered the vehicle and half were administered NA may have accounted for some of the variation observed between the two feeding periods, all mares were fed vehicle first followed by the NA supplement because we reasoned that giving the NA first may alter the environment of subsequent waves of follicles beyond the 2-week washout period, which could not be accounted for. Aspirating cohorts of different sized follicles in a crossover study would also provide additional insights into the uptake of NA by the ovaries. Given that NA supplementation elevated the concentration of NMN in the FF of the dominant follicle, the concentration at which improvements in oocyte developmental competence would be observed remains unknown in mares, and future studies are needed to assess the quality of oocytes collected from NA-fed mares.

## 5. Conclusions

This study revealed that supplementing the diet of mares with NA at a dose of 3 g daily during the follicular phase of the oestrous cycle altered the NA metabolite composition of the dominant follicle. Without NA supplementation, the level of NMN was significantly lower in FF than in the corresponding plasma. In contrast, with NA supplementation, the concentration of NMN in FF was increased 4-fold compared with that in plasma. Given that supplementation of NAD^+^ precursors, particularly NMN, has been found to enhance oocyte quality and embryo development in other species, it is tempting to propose that the observed changes in the follicular environment may translate to improved oocyte viability and mare fertility. The findings of this study will inform future investigations into the effects of orally administered NA on equine oocyte developmental competence and provide insights into the use of this dietary intervention as a strategy to improve reproductive outcomes in older mares.

## Figures and Tables

**Table 1:** The concentrations (ng/µL) of NAD^+^ precursors in the plasma and follicular fluid (FF) of mares fed the vehicle and nicotinic acid (NA) treatments. Values are presented as the means ± sem. Within each row, values labelled with the same superscript letter are significantly different.

Metabolite	Vehicle Treatment			NA Treatment		
	Plasma Prior to First Feed *	Plasma at FF Aspiration ^†^	FF	Plasma Prior to First Feed *	Plasma at FF Aspiration ^†^	FF
NA	19.9 ± 0.5	57.3 ± 21.5	22.0 ± 4.6 ^a^	99.2 ± 55.7	70.8 ± 15.4	1043.9 ± 953.5 ^a^
NAM	149.7 ± 16.1 ^a^	200.3 ± 20.9	96.5 ± 38.4 ^b^	1581.3 ± 416.9 ^a^	875.6 ± 361.1	1641.7 ± 256.1 ^b^
NaAD	6.6 ± 0.8 ^a^	4.6 ± 1.0 ^b^	2.0 ± 0.8	0.7 ± 0.3 ^a^	0.9 ± 0.3 ^b^	0.3 ± 0.2
NMN	9.5 ± 2.4 ^a^	5.7 ± 1.3	1.5 ± 0.5 ^b^	2.4 ± 0.4 ^a^	3.3 ± 0.9	15.0 ± 6.5 ^b^
NUR	3.6 ± 0.3 ^a^	2.6 ± 0.2	2.1 ± 0.2	0.0 ± 0.0 ^a^	0.6 ± 0.2	5.5 ± 5.1
NaR	2082.1 ± 761.4	3363.4 ± 952.9	4564.4 ± 1635.2	3843.8 ± 1823.3	8408.5 ± 2700.4	3363.3 ± 2094.3
NR	12161.5 ± 1490.8 ^a^	11653.5 ± 1417.0 ^b^	10871.6 ± 721.6 ^c^	2029.6 ± 97.9 ^a^	2073.5 ± 195.3 ^b^	1858.0 ± 51.9 ^c^

NA: Nicotinic Acid; NAM: Nicotinamide; NaAD: Nicotinic Acid Adenine Dinucleotide; NMN: Nicotinamide Mononucleotide; NUR: Nicotinuric Acid; NaR: Nicotinic Acid Riboside; NR: Nicotinamide Riboside. * Blood samples were collected immediately prior to the first vehicle or NA treatment. ^†^ Blood samples were collected immediately prior to sedation for follicle aspiration, approximately 16–18 h after the last vehicle or NA treatment.

## Data Availability

The data that support this study will be shared upon reasonable request to the corresponding author.
